# Longitudinal study of alexithymia and multiple sclerosis

**DOI:** 10.1002/brb3.194

**Published:** 2013-12-18

**Authors:** Khadija Chahraoui, Céline Duchene, Fabien Rollot, Bernard Bonin, Thibault Moreau

**Affiliations:** 1 Laboratoire de Psychopathologie et de Psychologie Médicale, Université de BourgognePôle AAFE, Dijon, France; 2 Service de Neurologie Clinique Bourguignonne de la Sclérose en Plaques, University HospitalDijon, France; 3 Service de Psychiatrie et d'Addictologie, University HospitalDijon, France

**Keywords:** Alexithymia, anxiety, depression, longitudinal study, multiple sclerosis

## Abstract

**Objective:**

The aim of this study was to investigate the course of alexithymia and its relation with anxiety and depression in patients with multiple sclerosis (MS), over a period of 5 years.

**Methods:**

Sixty-two MS patients were examined at two timepoints, 5 years apart, and they answered questionnaires collecting socio-demographic, medical, and psychological data (depression, anxiety, alexithymia).

**Results:**

Our data show that emotional disorders remain stable over time in patients with MS, particularly as regards alexithymia and anxiety. Conversely, the rate of depression decreased between the two evaluations, falling from 40% to 26%. The two dimensions of alexithymia (i.e., difficulty describing and difficulty identifying feelings) were correlated with anxiety and depression, whereas the third component of alexithymia (externally oriented thinking) was independent, and was the only component to change over time, with a significant fall observed at 5 years.

**Conclusion:**

Alexithymia was associated with increased severity of anxiety and attack relapses.

## Introduction

Numerous reports in the literature have previously underlined the importance of mood disturbances and emotional manifestations associated with evolving multiple sclerosis (MS) (Feinstein and Feinstein 2001; Mohr and Cox 2001; Montreuil and Petropoulou 2003; Montel and Bungener 2007; Dahl et al. 2009). Indeed, lifetime prevalence of major depressive disorder in MS patients is estimated to be around 50% (Mohr and Cox 2001). Although less attention has been focused on anxiety, it is also reported to be common, affecting an estimated 34% of patients (Montel and Bungener 2007). Other emotional disturbances have been reported in patients with MS, such as pathological laughing and crying, emotional lability, and alexithymia (Montreuil and Petropoulou 2003; Montel and Bungener 2007).

Alexithymia is a psychological construct initially described by Sifneos (1973) based on speech patterns from patients with classic psychosomatic diseases. It was subsequently also observed among patients with a variety of psychiatric disorders involving disturbances in emotional regulation (Guilbaud et al. 2002). Alexithymia comprises four main aspects: first, difficulty in identifying and describing feelings to others; second, restricted imaginative processes; third, a propensity to act in order to avoid resolving conflicts; and fourth, a detailed description of facts, events, and physical symptoms (Taylor 1984, 2000). Among the scales used to measure alexithymia, the Toronto Alexithymia Scale (TAS-20; Bagby et al. 1994) evaluates three main aspects (Dahl et al. 2009): (1) difficulties identifying feelings (DIF); (2) difficulties describing feelings (DDF); and (3) externally oriented thinking (EOT). The first two factors refer to emotional awareness and expression and might therefore be considered as “affect-related,” while the third factor refers to a specific tendency to deal with superficial themes and to avoid affective thinking and may therefore be considered more cognitive (Grynberg et al. 2010).

In patients with MS, alexithymia is mainly characterized as difficulty in identifying and describing emotions, a paucity of fantasies (e.g. lack of daydreams or dreams), and a discourse centered on facts and symptoms (Montreuil and Lyon-Caen 1993). Alexithymia may play a role in the development and severity of depression (Bodini et al. 2008; Gay et al. 2010). Indeed, MS ultimately leads to significant limitations and loss of autonomy due to the evolving nature of the disease, and these changes can require considerable and repeated social, professional, and familial adjustments. The profound changes and progressive loss of autonomy can create negative emotions and painful psychological experiences (Montreuil and Petropoulou 2003). Therefore, the patient affected by MS must necessarily work through a mourning period in order to be able to assimilate these losses in psychological terms (Jose 2008). Alexithymia could therefore be a major factor of vulnerability in this respect in that it contributes to the inhibition of emotional expression and of the capacity to mentalize the psychic trauma associated with the disease and its course. Alexithymia may also represent a key psychological factor that hampers true emotional and cognitive integration of the changes related to the disease.

Alexithymia in patients with MS has been investigated by only a few studies from France and elsewhere (Montreuil and Lyon-Caen 1993; Pelletier et al. 2000; Chahraoui et al. 2008; Gay et al. 2010). Studies using the TAS-20 with the French cutoff (clinical threshold of >55) found that prevalence of alexithymia is estimated to be between 40% and 50% in the MS population (Montreuil and Lyon-Caen 1993; Chahraoui et al. 2008). An Italian study that used the North American cutoff value for determining the presence of alexithymia (i.e., >60) observed a prevalence of 13.8% in a sample of 58 patients (Bodini et al. 2008). Another study from France found a rate of 23.2% among a sample of 115 patients with the same cutoff values (Gay et al. 2010).

It should be noted that alexithymia can represent either a stable personality trait that conditions an inappropriate reaction to stress (Sifneos 1973), or alternatively, a factor secondary to stressful situations. In this latter case, alexithymia would then serve a defensive purpose as a means of coping (Parker et al. 1998). Studies on the topic have been unable to investigate this distinction to date as they have been purely descriptive. However, it would appear that the relation between the state and trait components is complex. Indeed, a study by Berthoz et al. (1999) reported that alexithymia is a multidimensional construct, with certain dimensions linked to personality traits, whereas others are linked to states.

It appeared timely and useful to us to perform a longitudinal study in order to improve our understanding of the changing profile of vulnerability over time linked to alexithymia in MS patients. To the best of our knowledge, no study to date has addressed this specific question. Few studies have evaluated the course of depression and anxiety in MS patients over several years, and available results have reported the relative stability of depression and anxiety over time (Schreurs et al. 2002; Arnett and Randolph 2006) in this population, albeit with some interindividual differences (Beal et al. 2007). In all these studies, clinical variables did not appear to play any major predictive role in the emotional changes observed over time (Beal et al. 2007). Several studies failed to find any relation between the course of anxiety or depression, and the duration of disease or form of MS (Galeazzi et al. 2005). Conversely, other authors reported that greater functional limitation was associated with greater depressive symptoms (Beal et al. 2007). Similarly, Giordano et al. (2011) recently reported that anxiety is greater at the beginning of the disease (time of diagnosis), and diminishes 6 months later.

In this context, the aim of this study was to investigate the course of alexithymia and its relation with anxiety and depression in patients with MS, over a period of 5 years. Improved knowledge of the course of emotional disturbances over time could help us to better understand how MS patients adapt to their handicap in psychological terms over time.

## Methods

### Study population

The study was approved by the Ethics Committee of the region of Burgundy (Comité de protection des personnes Est-I). Patients suffering from MS and treated at the Burgundy MS Clinic (Clinique Bourguignonne de la Sclérose en Plaques) or at Dijon University Hospital (CHU de Dijon) were eligible for inclusion over a period of 14 months (January 2005–March 2006). All patients with a confirmed diagnosis of MS who had regular follow-up consultations scheduled during the inclusion period at either of the two participating centers were contacted by post prior to their consultation with information about the study. Patients who agreed to participate were seen by a psychologist after their medical consultation for the initial visit (Timepoint 1, T1). During this visit, baseline variables were recorded, the three measurement instruments for the study were administered, and the patients had an individual interview with the psychologist to describe their experience of their disease. All patients who participated in T1 were contacted by letter or by phone 5 years later (Timepoint 2, T2) for follow-up evaluation, comprising a second administration of the three measurement instruments and an interview with the psychologist.

Subjects were definitively included after providing written informed consent.

### Measures

We recorded socio-demographic variables (age, gender, level of education, marital status, number of children, profession, invalidity status), medical data (duration of disease, clinical form of MS, number of attacks, treatment), and history of psychiatric disorders (psychiatric disorders requiring hospitalization, treatment, or consultation with a psychiatrist).

Level of physical handicap was assessed with the widely used Expanded Disability Status Scale (EDSS) (Kurtzke 1983), administered by a neurologist. The EDSS quantifies disability in MS patients based on eight functional systems or parameters, of which four are major (pyramidal, cerebellum, sensory, and brainstem) and four are minor (bowel and bladder, visual, mental, and others). An ordinal clinical rating ranging from 0 to 6 or 7 is given for each functional system, corresponding to increasing severity. The global EDSS score is an ordinal scale ranging from 0 (no disability) to 10 (death due to MS) in half-point increments.

Depression was evaluated using the abridged Beck Depression Inventory (BDI; Beck and Beamesderfer 1974). This self-administered questionnaire comprises 13 items and investigates the severity of depression, particularly its subjective aspects. BDI is more prone to assess “state-depression” and it is also indicated for the evaluation of depression associated with MS because there are few references to the somatic component. Each item is composed of four statements corresponding to four degrees of increasing intensity of a symptom (from 0 to 3). The overall score is obtained by summing the scores of the 13 individual items. The overall score can range from 0 to 39. A score between 8 and 15 indicates moderate depression, and a score >16 indicates severe depression.

Anxiety was evaluated by means of Spielberger's STAI (the State-Trait Anxiety Inventory), which is one of the most frequently used anxiety self-evaluation scales internationally, in particular, in the medical field (Spielberger 1983). It consists of two separate parts that independently evaluate trait anxiety and state anxiety. Each part contains 20 items subdivided into four levels as a function of their intensity or frequency. The trait anxiety scale is used to evaluate personality-related anxiety characteristics, while the state anxiety scale makes it possible to measure the changes induced by various experimental situations. In this study, we used trait anxiety. According to the French adaptation of this instrument (Bruchon-Schweitzer and Paulhan 1993), a score between 46 and 55 indicates a moderate level of anxiety, a score between 56 and 65 a high level of anxiety, and a score >66 indicates a very high level of anxiety.

Alexithymia was evaluated using the 20-item TAS-20 (Loas and Fremaux 1995). This self-administered questionnaire evaluates three main clinical facets of alexithymia, namely difficulty identifying feelings, difficulty describing feelings, and EOT (Zech et al. 1999). In order to facilitate comparison with other studies, we assessed the presence or absence of alexithymia using internationally accepted cutoff values, as follows: 20–50°: nonalexithymic patients; 51–60°: borderline alexithymic patients; 61–100°: alexithymic patients. These thresholds are validated for use with the French-language version of the instrument (Loas et al. 1996; Gay et al. 2010).

### Statistical analysis

Data were analyzed using SPSS version 16 (IBM Corp, Armonk, NY). First, descriptive analysis was performed on data recorded at T1 and T2. The Wilcoxon test (for quantitative variables) and the Mac Nemar test (for categorical variables) were used as appropriate to compare subjects’ results on the different questionnaires at T1 and T2.

The relationship between alexithymia and the other variables (clinical, demographic, and psychological variables) was investigated using Spearman's correlation coefficient and subsequently by multiple stepwise logistic regression analysis in order to estimate the contribution of several clinical variables to the presence of alexithymia. A *P*-value <0.05 was considered statistically significant.

## Results

At T1, 62 patients (21 men, 41 women) participated, age 20–77 years. At T2 (5 years later), 44 patients participated (14 men, 30 women), 13 had been lost to follow-up, 4 refused to participate, and 1 patient had died. The proportion of male to female participants is in line with the gender distribution of MS (2:1 for women:men; Kingwell et al. 2013). The majority of participants were living maritally (69.4% at T1 and 77.3% at T2).

In clinical terms, patients primarily presented relapsing-remitting MS (80.64% at T1 and 68.18% at T2). The average duration of disease was 10.92 years at T1, and the average degree of handicap was 3.07 at T1 and 3.83 at T2. In total, 59.7% of participants were professionally active at T1, and 56.8% at T2. The demographic and clinical characteristics of the study population are presented in Table 1.

**Table 1:** Demographic and clinical characteristics of the study population at timepoints 1 and 2.

	Time 1 (*n* = 62)	Time 2 (+5 years) (*n* = 44)
Age, mean (SD)	41.37 (10.86)	46.17 (11.32)
Female sex	41 (66.1)	30 (68.1)
Married, *n* (%)	43 (69.4)	34 (77.3)
Professionally active, *n* (%)	37 (59.7)	25 (56.8)
Disease course		
Relapsing-remitting, *n* (%)	50 (80.64)	30 (68.18)
Primary/secondary progressive, *n* (%)	12 (19.35)	14 (31.82)
Disease duration (years), mean (SD)	10.92 (8.66)	16.52 (8.19)
Relapses, mean (SD)	5.53 (4.52)	7.59 ± 4.66
EDSS Score, mean (SD)	3.07 (2.20)	3.83 (2.36)

SD, standard deviation; EDSS, expanded disability status scale.

Table 2 shows the frequency of alexithymia, depression, and anxiety at T1 and T2. At T1, we observed 38.7% nonalexithymic patients; 30.6% borderline alexithymic patients and 30.6% alexithymic patients. These proportions did not differ significantly between T1 and T2 (Table 2).

**Table 2:** Frequency of depression, anxiety, and alexithymia at timepoints 1 and 2.

	Time 1 (*n* = 62)	Time 2 (*n* = 44)
Alexithymia (TAS-20)		
No alexithymia, *n* (%)	24 (38.7)	17 (38.6)
Borderline alexithymia, *n* (%)	19 (30.6)	14 (31.8)
Alexithymia, *n* (%)	19 (30.6)	13 (29.5)
Anxiety (STAI-T)		
Moderate anxiety, *n* (%)	15 (24.2)	11 (25)
High anxiety, *n* (%)	12 (19.4)	9 (20.5)
Depression (BDI)		
Moderate depression, *n* (%)	20 (32.3)	10 (22)
Severe depression, *n* (%)	5 (8.1)	2 (4.9)

TAS-20, 20-item Toronto Alexithymia Scale; STAI-T, State-Trait Anxiety Inventory – trait scale; BDI, Beck Depression Inventory.

Moderate or severe anxiety was observed in 27 patients (34.6%) at T1 and 20 (45.5%) at T2 and no significant difference was observed between T1 and T2.

Conversely, there was a significant reduction in the proportion of patients presenting depression (moderate or severe) at T2 versus T1 (*P* = 0.02 by the MacNemar test). Accordingly, 25 patients (40.4%) had moderate to severe depression at T1 and 12 (26.9%) at T2.

Patient scores from the different questionnaires administered at T1 and T2 are shown in Table 3. The overall depression score decreased significantly between T1 and T2 (*P* = 0.01), while the scores for anxiety and alexithymia remained stable, with the exception of the “EOT” factor of alexithymia, which decreased significantly between timepoints (*P* = 0.005). We also observed a small increase in EDSS score, indicating a slight progression of the level of handicap in these patients after 5 years (+0.76).

**Table 3:** Changes in overall patient scores for depression, anxiety, and alexithymia between timepoints 1 and 2.

	Time 1 (*N* = 62), Mean ± SD	Time 2 (*N* = 44), Mean ± SD	*Z*-value/*P*-value^1974^
EDSS score	3.07 (2.2)	3.83 (2.36)	−2.18/0.02
Depression (BDI)	7.31 (6.15)	5.95 (5.33)	−2.49/0.01
Anxiety (STAI)	44.76 (11.97)	45.89 (11.82)	−0.02/0.98
Alexithymia total score (TAS-20)	53.13 (11.24)	53.18 (12.67)	−0.86/0.38
Difficulty identifying feelings factor	18.84 (6.29)	18.89 (6.43)	−0.52/0.60
Difficulty describing feelings factor	14.37 (4.47)	14.64 (4.41)	−0.03/0.97
Externally oriented thinking factor	19.89 (4.40)	17.86 (4.68)	−2.82/0.005

EDSS, expanded disability status scale; BDI, Beck Depression Inventory; STAI, State-Trait Anxiety Inventory; TAS-20, 20-item Toronto Alexithymia Scale.

Wilcoxon test.

While overall scores for alexithymia and anxiety did not change significantly between T1 and T2, we did note interindividual differences in scores between the two timepoints (Table 4). Evaluation of each patient's position with regard to the threshold value revealed that two thirds of patients maintained a stable score over time for anxiety and alexithymia, whereas approximately one third of patients changed category, either moving above or below the threshold over time (Table 4). Accordingly, scores decreased to levels below the threshold value in 29.5% for depression, 22.7% for anxiety, and 18.2% for alexithymia. Scores increased to values above the threshold in 9.1% for depression, 15.9% for anxiety, and 15.9% for alexithymia (Table 4).

**Table 4:** Change in depression, anxiety, and alexithymia scores between timepoints 1 and 2.

	Score unchanged	Decreased	Increased
Depression (BDI)	27/44, 61.4%	13/44, 29.5%	4/44, 9.1%
Anxiety (STAI-T)	27/44, 61.4%	10/44, 22.7%	7/44, 15.9%
Alexithymia (TAS-20)	29/44, 65.9%	8/44, 18.2%	7/44, 15.9%

BDI, Beck Depression Inventory; STAI-T, State-Trait Anxiety Inventory – trait scale; TAS-20, 20-item Toronto Alexithymia Scale.

We compared the clinical characteristics between these groups and noted only two significant differences: In patients whose depression increased at T2, there was also a significant increase in anxiety score (on average +17.5; *P* = 0.0001). Second, patients whose anxiety score decreased to subthreshold values at T2 also had significantly lower scores on the “difficulty describing feelings” dimension of the TAS-20 (11.7 vs. 15.67 and 14.7 in the other two groups, *P* = 0.04).

Finally, overall comparisons between T1 and T2 were not affected by the high dropout rate observed between T1 and T2 (29%). Indeed, mean scores calculated for patients who completed the study at both timepoints were not significantly different from the mean scores in the initial population of 62 patients.

The relationships between alexithymia, and medical and psychological variables were analyzed using Spearman's correlation coefficient (Table 5). No significant correlation was observed between alexithymia and any of the demographic or clinical variables recorded. Alexithymia scores were mainly positively correlated with anxiety and depression at T1 and T2 (Table 5). The subscales “difficulty identifying feelings” and “difficulty describing feelings” were also significantly correlated with anxiety (*r* = 0.445, *P* < 0.01, and *r* = 0.499, *P* < 0.001, respectively) and depression (*r* = 0.279, *P* < 0.01, and *r* = 0.399, *P* < 0.007, respectively). Conversely, the “EOT” factor was not significantly correlated with either anxiety or depression, but was correlated with the number of relapses at T2 (*r* = 0.31, *P* = 0.01).

**Table 5:** Correlations between alexithymia, depression, and anxiety scores at timepoints 1 and 2.

	1	2	3	4	5	6
1. Alexithymia (TAS-20) – Time 1	–	0.398**^1993^	0.272*^1993^	0.253	0.283	0.242
2. Anxiety (STAI) – Time 1	0.398**^1993^	–	0.696**^1993^	0.340*^1993^	0.634**^1993^	0.504**^1993^
3. Depression (BDI) – Time 1	0.272*^1993^	0.696**^1993^	–	0.412**^1993^	0.567**^1993^	0.555**^1993^
4. Alexithymia (TAS-20) – Time 2	0.253	0.340*^1993^	0.412**^1993^	–	0.433**^1993^	0.387**^1993^
5. Anxiety (STAI) – Time 2	0.283	0.634**^1993^	0.567**^1993^	0.433**^1993^	–	0.850**^1993^
6. Depression (BDI) – Time 2	0.242	0.504**^1993^	0.555**^1993^	0.387**^1993^	0.850**^1993^	–

TAS-20, 20-item Toronto Alexithymia Scale; STAI-T, State-Trait Anxiety Inventory – trait scale; BDI, Beck Depression Inventory.

*P < 0.05; **P < 0.01.

Multivariate stepwise logistic regression analysis identified anxiety and the number of relapses as being significantly related to the presence of alexithymia at T2 (Anxiety: *R*^2^* *=* *0.20, *F *=* *12.10, *β *=* *0.47, *t *=* *3.47, *P* = 0.001; Number of relapses: *R*^2^* *=* *0.38, *F *=* *14.25, *β *=* *0.44, *t *=* *3.60, *P* = 0.001).

## Discussion

To the best of our knowledge, this is the first study to investigate alexithymia in MS over time. In this study, we observed a prevalence of around 30% of alexithymia in our population of patients with MS, using the TAS-20 cutoff values, and this proportion remained stable over the two timepoints studied. We chose to use international cutoff values, and not French values (Loas and Fremaux 1995) in this study so that our results could more easily be compared with other reports. This prevalence is in line with that reported by Gay et al. (2010) in another French population of MS patients. Conversely, our results do not concord with those of Bodini et al. (2008) who reported a lower prevalence, at only 13.8% in a population of patients from Italy. The discrepancies could likely be explained by the different clinical characteristics of our respective samples (e.g., lower level of handicap and higher rate of male patients in Bodini's study), or by cultural differences, or other factors that remain to be elucidated.

As previously reported by several authors (Bodini et al. 2008; Chahraoui et al. 2008; Gay et al. 2010), we observed a relation between alexithymia and both anxiety and depression, and at both timepoints of this study. The rates of anxiety and depression were consistently high in this study, with around 40% of MS patients suffering from anxiety problems at both T1 and T2, and this finding was stable over time. Conversely, the rate of depression tended to decrease between the two evaluations, falling from 40% to 26%. Multivariate logistic regression showed that alexithymia seems to be more strongly associated with anxiety. These results underline the similar manner in which alexithymia and anxiety are associated, as well as the stability of these disorders over time. The great emotional difficulty experienced by patients with MS has consistently been reported in the literature (Feinstein et al. 1999; Dahl et al. 2009) and the persistence of emotional disturbances over time has previously been highlighted by other authors (Arnett and Randolph 2006; Beal et al. 2007). It is possible that this persistence arises from a permanent incapacity of these patients to cope with the disease, particularly as prognosis is very uncertain in terms of progression of handicap (Giordano et al. 2011). The unpredictable nature of the progression of MS appears to be a central component in understanding the persistence of the emotional problems. Indeed, MS is characterized by the occurrence of attacks (relapses) of worsening neurological function that are highly unpredictable, and the patient cannot anticipate either the occurrence of an attack, or the type or intensity of symptoms. Predicting disease progression is, therefore, a dimension of MS that is extremely challenging. From a clinical point of view, some patients may experience several relapses in the same year, whereas others may go 10 years without an attack. Furthermore, while some attacks can have more or less severe effects that may partially or totally recede, others may herald a functional deficit, such as impaired motor function that can remain and become permanent. While the relapsing-remitting form of MS highlights, in particular, the uncertainty experienced by patients with MS (Montreuil and Lyon-Caen 1993), the progressive form, with its slowly but constantly worsening neurological function, also leaves patients feeling insecure and uncertain about their future, once the symptoms or irreversible deficits begin to appear. The long-term course of MS is characterized by ambiguity, which can lead to a real fear of what lies ahead, and a permanent state of anxiety (Montreuil and Lyon-Caen 1993; Mohr and Cox 2001). Patients are in a constant state of worry; when they are well, they worry that they may soon suffer a relapse, and when they do suffer an attack, they worry that it may the beginning of a rapid decline, or the harbinger of further deficits. Furthermore, MS attacks can be experienced as a veritable traumatic event by patients, as onset is often sudden and unexpected, which can be complex and painful to cope with and accept (Jose 2008). Many patients find it hard to let go of their hopes of living normally, and accept the physical constraints imposed on them by their disease. The wide gap between what they are physically able to do, and what they would like to be able to do, is often hard to accept.

Another noteworthy point is that only two dimensions of alexithymia, namely difficulty describing and difficulty identifying feelings, were correlated with anxiety and depression, whereas the third component of alexithymia (EOT) is independent of both these disorders. We also observed that this latter factor was the only one to evolve over time, with a significant fall in this dimension observed at 5 years. It is also the only factor to be specifically correlated with the number of MS relapses. Given that EOT is not correlated with either anxiety or depression, it is possible that it may be a form of defensive strategy for coping with the traumatic experience of MS relapses. Accordingly, by orienting their preoccupations and thoughts externally, the patient is able to avoid facing up to their interior feelings, and more particularly, the anxiety arising from the traumatic nature of the course of the disease. We could even go so far as to hypothesize that EOT may represent a form of avoidance and denial of reality employed by the patient to protect themselves against excessively distressing feelings.

The fact that the effect of this factor decreases over time could suggest more successful adaptation to the disease, in so far as the patient has less need to use this defensive strategy. This is line with the reduction in depression over time, which may also indicate better adjustment to disease after a number of years. These findings are in line with the study by Chwastiak et al. (2002), who reported that depressive symptoms decreased in the long-term after diagnosis. The question arises, therefore, as to whether the reduction in depression over time can be explained by better adjustment to the different disease-related handicaps, and by improved coping strategies that allow the patient to adapt better, thereby reducing depression.

However, our results cannot be extrapolated to all patients with MS, as the population included in this study presented a mild to moderate level of handicap (EDSS: 3.83 ± 2.36), thus limiting applicability of our findings to other groups with a similar profile. Our results should thus be interpreted in the context of the clinical characteristics of our study population. Indeed, the level of handicap observed in our population suggests that these patients were able to maintain active social and professional lives. Indeed, 56% of the patients in this study were still professionally active at T2, which could be considered to protect them to a certain extent by maintaining a network of social contact and support, whose positive effects on MS have previously been documented (Gay et al. 2010).

In conclusion, our findings suggest that there is a certain level of stability in emotional disorders over time in patients with MS particularly as regards alexithymia and anxiety, which are inextricably related, and likely due to the unpredictable course of the disease. However, it is also important to investigate interindividual differences. Indeed, although two thirds of our study population maintained a stable profile over time, the remaining third evolved (either for better or for worse) over the 5 years between evaluations. It is, therefore, important to investigate further the emotional disturbances occurring in the context of MS in order to identify protective factors, and factors (psychological, biological, genetic, or social) that may render patients more vulnerable to emotional problems, with a view to developing personalized strategies for accompanying patients with MS.

## References

[b1] Arnett PA, Randolph JJ (2006). Longitudinal course of depression symptoms in multiple sclerosis. J. Neurol. Neurosurg. Psychiatry.

[b2] Bagby RM, Parker JD, Taylor GJ (1994). The twenty-item toronto alexithymia scale–I. Item selection and cross-validation of the factor structure. J. Psychosom. Res.

[b3] Beal CC, Stuifbergen AK, Brown A (2007). Depression in multiple sclerosis: a longitudinal analysis. Arch. Psychiatry Nurs.

[b4] Beck AT, Beamesderfer A, Pichot P (1974). Assessment of depression: the depression inventory. Psychological measurements in psychopharmacology (modern problems of pharmacopsychiatry).

[b5] Berthoz S, Consoli S, Perez-Diaz F, Jouvent R (1999). Alexithymia and anxiety: compounded relationships? A psychometric study. Eur. Psychiatry.

[b6] Bodini B, Mandarelli G, Tomassini V, Tarsitani L, Pestalozza I, Gasperini C (2008). Alexithymia in multiple sclerosis: relationship with fatigue and depression. Acta Neurol. Scand.

[b7] Bruchon-Schweitzer M, Paulhan I (1993). Manuel de l'inventaire d'anxiété état-trait forme Y (STAI-Y) (adapt. française).

[b8] Chahraoui K, Pinoit JM, Viegas N, Adnet J, Bonin B, Moreau T (2008). Alexithymia and links with depression and anxiety in multiple sclerosis. Rev. Neurol. (Paris).

[b9] Chwastiak L, Ehde DM, Gibbons LE, Sullivan M, Bowen JD, Kraft GH (2002). Depressive symptoms and severity of illness in multiple sclerosis: epidemiologic study of a large community sample. Am. J. Psychiatry.

[b10] Dahl OP, Stordal E, Lydersen S, Midgard R (2009). Anxiety and depression in multiple sclerosis. A comparative population-based study in Nord-Trondelag County, Norway. Mult. Scler.

[b11] Feinstein A, Feinstein K (2001). Depression associated with multiple sclerosis. Looking beyond diagnosis to symptom expression. J. Affect. Disord.

[b12] Feinstein A, O'connor P, Gray T, Feinstein K (1999). The effects of anxiety on psychiatric morbidity in patients with multiple sclerosis. Mult. Scler.

[b13] Galeazzi GM, Ferrari S, Giaroli G, Mackinnon A, Merelli E, Motti L (2005). Psychiatric disorders and depression in multiple sclerosis outpatients: impact of disability and interferon beta therapy. Neurol. Sci.

[b14] Gay MC, Vrignaud P, Garitte C, Meunier C (2010). Predictors of depression in multiple sclerosis patients. Acta Neurol. Scand.

[b15] Giordano A, Granella F, Lugaresi A, Martinelli V, Trojano M, Confalonieri P (2011). Anxiety and depression in multiple sclerosis patients around diagnosis. J. Neurol. Sci.

[b16] Grynberg D, Luminet O, Corneille O, Grezes J, Berthoz S (2010). Alexithymia in the interpersonal domain: a general deficit of empathy?. Personality Individ. Differ.

[b17] Guilbaud O, Loas G, Corcos M, Speranza M, Stephan P, Perez-Diaz F (2002). Alexithymia in addictive behaviors and in healthy subjects: results of a study in French speaking subjects. Ann. Med. Psychol.

[b18] Jose SAM (2008). Psychological aspects of multiple sclerosis. Clin. Neurol. Neurosurg.

[b19] Kingwell E, Marriott JJ, Jette N, Pringsheim T, Makhani N, Morrow SA (2013). Incidence and prevalence of multiple sclerosis in Europe: a systematic review. BMC Neurol.

[b20] Kurtzke JF (1983). Rating neurologic impairment in multiple sclerosis: an expanded disability status scale (EDSS). Neurology.

[b21] Loas G, Fremaux D, Marchand MP (1995). Factorial structure and internal consistency of the French version of the twenty-item Toronto Alexithymia Scale in a group of 183 healthy probands. Encephale.

[b22] Loas G, Otmani O, Fremaux D, Lecercle C, Duflot M, Delahousse J (1996). External validity, reliability and basic score determination of the Toronto Alexithymia Scales (TAS and TAS-20) in a group of alcoholic patients. Encephale.

[b23] Mohr DC, Cox D (2001). Multiple sclerosis: empirical literature for the clinical health psychologist. J. Clin. Psychol.

[b24] Montel S, Bungener C (2007). Mood and emotional disorders in multiple sclerosis: a literature review. Rev. Neurol. (Paris).

[b25] Montreuil M, Lyon-Caen O (1993). Thymnic troubles and relation between alexithymia and inter-hemispheric dysfunction in multiple sclerosis. Rev. Neuropsychol.

[b26] Montreuil M, Petropoulou H (2003). Emotional disturbances in neurology and psychiatry: mood and emotions in multiple sclerosis. Neuropsy. News.

[b27] Parker JD, Taylor GJ, Bagby RM (1998). Alexithymia: relationship with ego defense and coping styles. Compr. Psychiatry.

[b28] Pelletier J, Benoit N, Montreuil M, Habib M (2000). Cognitive and emotional disorders in multiple sclerosis. Can a management strategy be envisioned?. Pathol. Biol. (Paris).

[b29] Schreurs KM, Bensing DT, de Ridder JM (2002). Fatigue in multiple sclerosis: reciprocal relationships with physical disabilities and depression. J. Psychosom. Res.

[b30] Sifneos PE (1973). The prevalence of “alexithymic” characteristics in psychosomatic patients. Psychother. Psychosom.

[b31] Spielberger CD (1983). State-trait anxiety inventory (form Y).

[b32] Taylor GJ (1984). Alexithymia: concept, measurement, and implications for treatment. Am. J. Psychiatry.

[b33] Taylor GJ (2000). Recent developments in alexithymia theory and research. Can. J. Psychiatry.

[b34] Zech E, Luminet O, Rimé B, Wagner H (1999). Alexithymia and its measurement: confirmatory factor analyses of the 20-item Toronto Alexithymia Scale and the Bermond–Vorst Alexithymia Questionnaire. Euro. J. Personality.

